# Assessing the Accuracy of Ancestral Protein Reconstruction Methods

**DOI:** 10.1371/journal.pcbi.0020069

**Published:** 2006-06-23

**Authors:** Paul D Williams, David D Pollock, Benjamin P Blackburne, Richard A Goldstein

**Affiliations:** 1 Department of Chemistry, University of Michigan, Ann Arbor, Michigan, United States of America; 2 Department of Biological Sciences, Biological Computation and Visualization Center, Louisiana State University, Baton Rouge, Louisiana, United States of America; 3 Division of Mathematical Biology, National Institute of Medical Research, Mill Hill, London, United Kingdom; University of Texas, United States of America

## Abstract

The phylogenetic inference of ancestral protein sequences is a powerful technique for the study of molecular evolution, but any conclusions drawn from such studies are only as good as the accuracy of the reconstruction method. Every inference method leads to errors in the ancestral protein sequence, resulting in potentially misleading estimates of the ancestral protein's properties. To assess the accuracy of ancestral protein reconstruction methods, we performed computational population evolution simulations featuring near-neutral evolution under purifying selection, speciation, and divergence using an off-lattice protein model where fitness depends on the ability to be stable in a specified target structure. We were thus able to compare the thermodynamic properties of the true ancestral sequences with the properties of “ancestral sequences” inferred by maximum parsimony, maximum likelihood, and Bayesian methods. Surprisingly, we found that methods such as maximum parsimony and maximum likelihood that reconstruct a “best guess” amino acid at each position *overestimate* thermostability, while a Bayesian method that sometimes chooses less-probable residues from the posterior probability distribution does not. Maximum likelihood and maximum parsimony apparently tend to eliminate variants at a position that are slightly detrimental to structural stability simply because such detrimental variants are less frequent. Other properties of ancestral proteins might be similarly overestimated. This suggests that ancestral reconstruction studies require greater care to come to credible conclusions regarding functional evolution. Inferred functional patterns that mimic reconstruction bias should be reevaluated.

## Synopsis

It is now possible to apply computational methods to known current protein sequences to recreate the sequences of ancestral proteins. By synthesising these proteins and measuring their properties in the laboratory, we can gain much information about the nature of evolution, better understand how proteins change and adapt over time, and develop insights into the environments of ancient organisms. Unfortunately, the accuracy of these reconstructions is difficult to evaluate. We simulate protein evolution using a simplified computational model and apply the various reconstruction methods to the sequences that arise from our simulations. Because we have the complete record of the evolutionary history, we can evaluate the reconstruction accuracy directly. We demonstrate that the reconstruction procedures in common use may have a bias toward overestimating the properties of these ancestral proteins, opposite to what has been assumed previously. An alternative method of creating these sequences is presented, Bayesian sampling, that can eliminate this bias and provide more robust conclusions.

## Introduction

With the development of extensive databases of genetic sequences and sophisticated phylogenetic inference methods, the reconstruction of putative ancestral genetic states is an increasingly common technique. Ancestral reconstruction has been used to estimate the properties of ancestral genomes [1], genes [2], and proteins [3–8]. These methods allow the study of adaptive selection, functional divergence, and evolutionary pathways, in a manner not possible without accurate knowledge of the past. Inferred protein sequences may be synthesised and experimentally characterised, allowing the deduction of historical conditions [6, 9]. The properties of the inferred ancestral protein can also be compared with the properties of modern proteins, leading to a better understanding of evolutionary processes, both for specific protein families and in general.

Increasingly sophisticated statistical methods have been developed to obtain more accurate inferences; two popular methods are maximum parsimony (MP) and maximum likelihood (ML). The MP method was the first used for ancestral protein sequence reconstruction [7] but is based on an extreme simplification of the evolutionary process. Because of this simplification, MP suffers from drawbacks such as the inability to resolve ambiguous, equally parsimonious reconstructions and an inability to provide statistically robust measures of confidence. A currently popular alternative method of reconstruction involves ML, where the ancestral states are chosen that represent the highest a posteriori probability at that position [10,11]. An advantage of the ML method is that it uses an explicit model of the substitution process, in contrast to the implicit model that lies behind MP approaches; the substitution model, however, is a phenomenological construct and does not provide a realistic description of the evolutionary process. A third approach, Bayesian inference (BI) is a more recent development in phylogenetic analysis [12–14] in which a quantity of interest is viewed as a posterior probability distribution, rather than a point or “best” estimate, as in MP and ML, and where an explicit summation over different possibilities for the nonessential parameters is performed. In particular, BI ancestral reconstruction methods have been developed that sample over trees, branch-lengths, and substitution models [15,16]. The idea of creating ancestral sequences by sampling amino acid residues over posterior distributions has not been popular, partly because it is counterintuitive to many people not to choose a best estimate but also because the use of BI would seem to require the synthesis and characterisation of multiple proteins. (To avoid confusion, it is important to note here that when we refer to MP, ML, and BI techniques, we are referring to the MP, ML, or BI approach to determining the ancestral state at each position in the protein; the choice of method for determining phylogenetic relationships is a separate issue.)

The accuracy of any ancestral reconstruction, and therefore the degree of confidence in the results, depends upon both the precision and the systematic error or bias in the reconstruction. Bias is particularly important to avoid, since it can easily lead to the appearance of spurious trends in functional inference. For example, an ML reconstruction study by Gaucher et al. [6] suggested that mesothermophilic, thermophilic, and hyperthermophilic bacteria all evolved from ancestral thermophiles, a conclusion based on the high thermostability of their reconstructed ancestral proteins. If ML reconstruction accurately estimates thermostability, the results of these studies are likely to be valid. In contrast, if statistical biases result in an overestimation of themostability, the conclusions made in this study may be incorrect, and if ML reconstructions underestimate thermostability, the conclusions may be even stronger than they at first appeared.

It has been suggested that reconstruction errors can be thought of as similar to random mutations; as random mutations are generally deleterious, reconstruction methods should have a bias toward reducing or eliminating the “performance” of a protein, presumably resulting in the underestimation of ancestral biophysical properties such as stability [8]. Although this assumption might be initially appealing to a casual reader, it is questionable whether a valid connection can be made between random mutations and errors in reconstructions, as the available variants are necessarily acceptable in a thermodynamic and functional context; the same cannot be said for random mutations.

How can this issue be addressed? Unfortunately, direct comparison of the results of ancestral reconstructions with the actual ancestral sequences is difficult. The recovery of ancestral DNA fragments from amber, ice, or peat has been limited, and susceptible to its own inaccuracies and time limitations. Known phylogenies and ancestors have been generated using artificial evolution experiments [17,18], but this approach has not yielded sufficient data for a detailed analysis of our question. Krishnan and colleagues [2] found that strong compositional trends suggested by both ML and MP reconstruction of primate mitochondrial DNA were not supported by BI, and were easily explained by methodological biases. In their study, inaccurate reconstructions were also shown to have potential deleterious effects on tRNA structure (and presumably function). Less has been done on the accuracy of protein reconstruction methods. As an alternative to direct comparison with known ancestral sequences, it is possible to perform in silico evolutionary simulations, enabling generation of larger data sets and more thorough testing of the reconstruction method, and we take that approach here.

Protein evolution occurs as an accumulation of random mutations in a polymorphic population, where the effect of various mutations may be different at different locations and may change over time, and where fixation probabilities depend upon the effects of the variants on the properties of the resultant proteins and upon the relative fitness of each variant in the population in which it arises. Ancestral reconstruction, conversely and necessarily, relies on phenomenological models of the sequence changes that occur as a result of this evolutionary process, where a diverse population is represented as a fixed sequence and locations are generally assumed to evolve independently with a pattern that is (with the exception of a distribution of absolute rates) the same for all locations at all time. In this paper, we simulate the evolution of a population of proteins as they undergo nearly neutral evolution with purifying selection, including the processes of divergence and speciation, based on a realistic phylogenetic tree obtained for a set of RNase A gene sequences. Proteins are modelled using an off-lattice framework and represented as sequences of 300 amino acid residues whose fitness depends on the probability of folding into an arbitrarily predetermined target structure. While the model of the protein thermodynamics is necessarily simplified, the model does capture the idea of stability being composed of a large number of small, multibody interactions distributed throughout the molecule. We then applied standard analytical tools based on current phenomenological models to perform the ancestral reconstruction of internal nodes using MP, ML, and BI approaches.

As we recorded the evolutionary process as it occurred, we were able to compare thermodynamic properties of the reconstructed proteins with those of the actual ancestors. As proteins were preequilibrated with respect to sequence, fitness, and composition before the simulations began, consistent biases in reconstruction were easily discerned as deviations from equilibrium properties. Although ML produces the most accurate sequences, the MP and ML methods result in significant errors in the estimation of the stability of ancestral proteins. Surprisingly, MP and ML generally *overestimate* thermodynamic stability. This counterintuitive result may be due to general statistical features of ancestral reconstruction, and is explained in detail below. Bayesian methods result in the smallest and most unbiased errors in stability, even when BI is used to produce a single sequence, with no increase in experimental effort. These results indicate that errors in reconstruction cannot be considered similar to random mutations and that the conclusions based on previous MP and ML reconstruction studies may be misleading and should be reevaluated.

## Results

Protein evolution was modelled in populations of 1,000 where fitness was based on the fraction of time that the protein would be folded in its target structure in thermodynamic competition with a large ensemble of competing structures. One hundred different simulations were performed, of which the results from 84 were analysed. After preequilibration, the proteins had an average stability of approximately −2.5 kcal/mol, corresponding to approximately 98.5% of the proteins being folded. As the maximum possible folded fraction is 100%, the fitness landscape at this point is close to flat, with mutational pressure due to the greater number of destabilising mutations compared to stabilising mutations counteracting any remaining selective pressure for increased stability. In other words, equilibration here means that the sequences were in mutation/selection balance, and there was no subsequent directional change in fitness as the simulations proceed. Subsequently, the stabilities of the evolving proteins were approximately constant; substitutions that slightly increased protein stability were generally preceded by or followed by substitutions that slightly decreased stability, as expected for nearly neutral evolution under purifying selection [19]. Amino acid composition of the evolving proteins was also approximately constant after preequilibration.

In each replication of the computational experiment, populations of proteins duplicated and diverged according to a phylogenetic tree reconstructed using RNase A sequences. The protein sequences from the terminal nodes of these simulations were used to reconstruct sequences at the internal nodes using MP, ML, and BI approaches. The substitution models and reconstructions were obtained using only the most common sequences at the end of the simulations, and although rates may vary, locations were assumed to evolve independently with a pattern that was the same for all locations at all times.

The ML method was the most accurate at estimating the true ancestral sequences on a site-by-site basis, with 93.7 ± 4.5% of all nodes at all sites correctly reconstructed. (A ± value represents the standard deviation of the accuracies of the ensemble of reconstructions. The uncertainty in the average accuracy is 0.4%.) This greater accuracy for ML is expected, since by definition ML chooses the most likely amino acid at every position. BI, which samples from the reasonably likely choices, was only slightly less accurate, with an average accuracy of 92.3 ± 5.2% (uncertainty in average accuracy of approximately 0.5%). MP, which does not include an explicit model of substitution, was less accurate than the other two methods, with an accuracy of 89.8 ± 6.9% (uncertainty in average accuracy of approximately 0.8%). Of the locations, 91.7% were predicted correctly both by ML and by BI. An additional 2.0% were predicted correctly by ML but not by BI, while only 0.6% of all locations were correctly predicted by BI but not by ML. Of the 5.6% predicted correctly by neither method, most of the time (4.3%) both methods made the same error. Overall, BI and ML predictions were identical for 96.0% of all locations.

Although it might be argued that MP is at a disadvantage, since the likelihood-based methods benefited from abnormally abundant and high-quality data to define the substitution model, it is also true that the model used is actually incorrect, since it takes neither site-specific model variation nor nonstationarity into account. This model inaccuracy is reflected in the difference between the posterior probabilities and the actual error rates. The most likely amino acids had an average posterior probability of 96.0%. (Distribution of posterior probabilities is shown in Figure S1.) The average expected error for the BI method for this dataset was 94.3%. Inaccuracy of the evolutionary model would also be expected for real proteins undergoing biological evolution, perhaps even more so, since matrices are often optimised on proteins other than the one under study, and the sizes of the datasets used to generate the model are usually smaller. As indicated in Table S1 and Figure S2), use of less accurate substitution models did not appreciably change the accuracy of the sequence reconstructions but generally increased the average posteriors. For instance, the use of a simple WAG substitution matrix [20] with default equilibrium frequencies and no gamma distribution resulted in a sequence accuracy of 93.4 ± 4.6% (uncertainty in average accuracy approximately 0.5%), only slightly worse than results obtained with the optimised model, but with a higher average posterior probability of 97.0%. (Obviously these trends would not continue for grossly inaccurate substitution models.) This highlights the fact that the average posterior only reflects the expected accuracy of the predictions *given* the accuracy of the model and that higher posterior probabilities do not indicate a better substitution model.

Since we recorded the evolutionary process as it occurred, we were also able to compare thermodynamic properties of the reconstructed proteins with those of the actual ancestors. Since the proteins were preequilibrated with respect to sequence, fitness, and composition before the simulations began, the central question was whether the variance and bias in reconstructed sequence properties were different from equilibrium values. Variances in reconstructed properties of individual ancestors were often greater than variance among ancestors, and biases were in some cases quite large and not in the direction predicted by naïve intuition.

**Figure 1:**
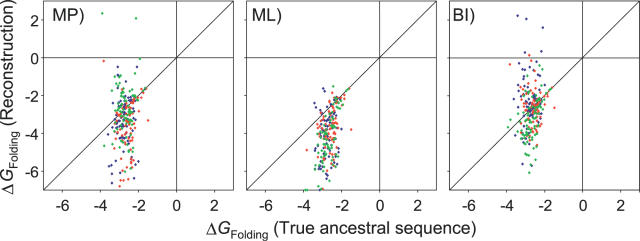
Stability (Represented as Δ*G*_Folding_) for the True Ancestor and Reconstructed Sequences Stability values shown for three nodes, as labelled in Figure S3: (A) shallow node, blue, (B) intermediate node, red, and (C) deep node, green. Each point represents the reconstruction of a single ancestral node in one of the 84 analysed simulations. Reconstructions were performed with MP, ML, and BI approaches. Δ*G*_Folding_ values represent the average of 100 reconstructions. Points on the diagonal represent reconstructions generating accurate ancestral protein stabilities.

Figure 1 compares the relative stabilities (Δ*G*_Folding_) for the true ancestral sequences with those of the reconstructions for three different ancestral nodes sampled at different tree depths. Here, the results from the BI analysis represent an average over 100 reconstructions for each of the 84 simulations. Given the relative accuracy of the various methods in reconstructing sequences, it is surprising to see that for predictions of thermodynamic stability, ML was the least accurate and BI the most accurate. Notice that the magnitude of the error can be quite large, and that the reconstructed stability values for the MP and especially for the ML reconstructions are generally substantially higher (Δ*G*_Folding_ more negative) than the true value. This indicates a strong systematic bias in ML and MP toward *overestimating* the protein stability. The results are for the most part qualitatively similar for each of the three nodes shown corresponding to different depths on the tree.

The random errors for the various reconstruction strategies are rather similar; the standard deviations of the errors in reconstruction stability are roughly comparable, with BI having the lowest random error (SD, 1.4 kcal/mol) followed by ML (SD, 1.6) and MP (SD, 1.8).

**Figure 2:**
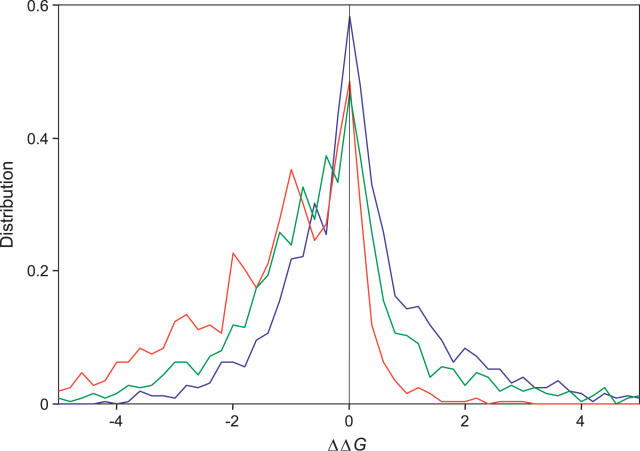
Distribution of Errors in Reconstruction Stabilities (ΔΔ*G*) When Reconstructions Are Made with MP (Green), ML (Red), and BI (Blue)

The tendency for MP and ML to substantially overestimate ancestral stability is demonstrated more comprehensively in Figure 2, which shows the overall distribution of ΔΔ*G* = Δ*G*_Folding_ (Reconstruction) − Δ*G*_Folding_ (True Ancestor) for the three different reconstruction methods. On average, MP reconstructions have Δ*G*_Folding_ values 0.4 ± 1.8 kcal/mol too negative and ML reconstructions have Δ*G*_Folding_ = 1.5 ± 1.6 kcal/mol too negative. The error in thermodynamic stability for the ML reconstructions was relatively insensitive to the substitution model used (Table S1). In comparison, Δ*G*_Folding_ for BI reconstructions are on average only 0.05 ± 1.4 kcal/mol too negative. (Again, ± values represent the range of the measurements for different ancestral nodes and simulations. The average errors have an uncertainty of ± 0.1 kcal/mol. Since ML has the lowest sequence error, BI slightly more, and MP by far the greatest, it is clear that errors in the ancestral sequence reconstruction do not translate directly to errors in the properties of the ancestral reconstructions.

ML shows a statistically significant (correlation coefficient −0.61, *p* = 0.02) increase in reconstruction bias with the depth of the node (measured as the shortest distance to an existent sequence); MP shows a near-significant increase (correlation coefficient −0.49, *p* = 0.06), while BI is not significantly statistically correlated with node depth (*p* = 0.49). Systematic errors in reconstruction produced by the three different methods show quite different dependencies on sequence error rate. ML methods produce a systematic bias that is roughly linear with sequence error, always overestimating protein stability. Sequence errors accumulate quickest in MP reconstructions, but beyond a sequence error rate of about 5%, reconstruction stability overestimates are roughly independent of sequence error. For sequence error rates below about 12%, BI slightly overestimates stability, but for higher sequence error rates, BI tends to substantially underestimate stability. It is not surprising that the Bayesian method eventually fails, since as reconstruction sequence error accumulates, more and more compensatory mutations at different sites will not be paired in any given reconstruction. At this level of divergence and reconstruction error, none of the methods provides a reliable reconstruction of ancestral stability.

**Figure 3:**
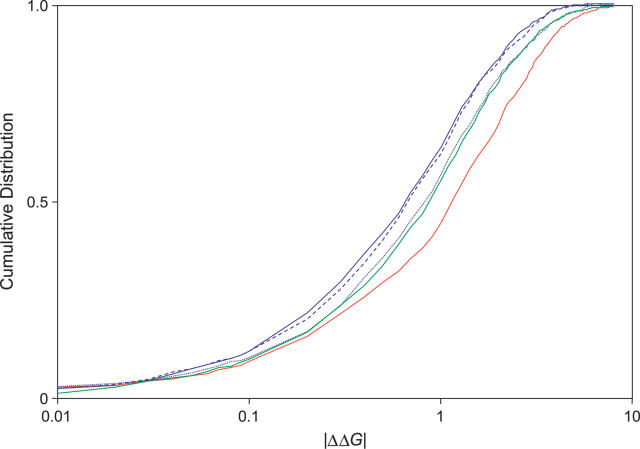
Cumulative Distribution of Absolute Errors in Reconstruction Stabilities (|ΔΔ*G*|) Colour code as in Figure 2. Error with BI reconstructions are shown when averaging is performed over 100 sequence reconstructions (solid line), ten sequence reconstructions (dashed line), and a single reconstruction (dotted line).

Figure 3 shows the cumulative distribution of absolute errors in reconstruction stabilities (|ΔΔ*G*|). Once again, there is clear evidence that BI results in smaller absolute errors than MP and ML. One disadvantage of BI, however, even for nodes where it is unbiased, is that it involves the reconstruction and recreation of a random sampling of sequences drawn from the a posteriori probability distribution. The data in Figures 1, 2, and 3 represent the average of 100 sequences drawn from the posterior amino acid distributions. Expressing, purifying, and characterising that number of sequences would be a daunting task. The question then arises, how quickly does BI degrade as the number of averaged sequences is reduced? How many sequences are required before the scatter due to sampling error is less than the other main sources of error? Figure 3 demonstrates that, in this case, there is only a small reduction in accuracy when only ten sequences are created and characterised, and that even creating a *single* ancestral sequence using BI outperforms ML and is comparable to MP in overall absolute error while maintaining a low systematic bias. Creating multiple sequences will lead to estimates with lower error.

## Discussion

The fundamental counterintuitive result of this study is that although ML ancestral reconstructions are closest to the true ancestral sequence, they are unreliable guides to ancestral thermodynamical properties. In contrast, BI reconstructions sampled from the posterior are on average slightly less similar to the ancestral sequence, but are a better reflection of ancestral properties, particularly when multiple samples are assayed. This result upholds an earlier prediction [2] that the same biases that lead to false reconstruction of cumulative sequence properties (i.e., nucleotide or amino acid frequencies) may also sometimes lead to false reconstruction of functional properties. In direct contrast to what has been suggested [8], MP and ML generally *overestimate* thermodynamic stability. BI results in the smallest and most unbiased errors in stability, even when BI is used to produce a single sequence, with no increase in experimental effort. This suggests that the results of previous MP and ML reconstruction studies may be misleading and might profitably be investigated for possible biases.

To understand why BI reconstructions are preferable, consider the different sources of error in the estimation of properties from recreated ancestral proteins. First, there are *random* errors in the creation of the ancestral sequences, that is, the discrepancies between the properties of the recreated sequence and the true ancestral sequences that average to zero over a large number of different instances. Second, there are the *systematic* errors, the discrepancies that cause a consistent trend toward underestimation or overestimation of the protein properties and do not average to zero. Finally, in the case of BI, there is the additional *sampling* error caused by estimating an average property value using only a small number of reconstructed sequences. The accuracy of this average will depend upon the number of reconstructions involved.

For our particular study, we find that the random errors for the various reconstruction strategies are rather similar; subtracting the (average) systematic bias, the standard deviations of the errors in reconstruction stability are roughly comparable, with BI having the lowest random error (SD, 1.4 kcal/mol) followed by ML (SD, 1.6) and MP (SD, 1.8). There is a much more significant difference in the systematic error, with BI having a much lower systematic error (〈ΔΔ*G*〉 = −0.05 ± 0.2 kcal/mol) compared with the ML (〈ΔΔ*G*〉 = −1.5 ± 0.2) and MP (〈ΔΔ*G*〉 = −0.4 ± 0.2) approaches. The BI reconstruction method has an associated sampling error, but this error is relatively small when ten reconstructions are sampled (SD, 0.4 kcal/mol). To make a different comparison, when ten reconstructions are sampled, the extra sampling error is roughly equal to the range of stability values observed at any given ancestral node in different simulations, a rough estimate of when further accuracy becomes unimportant for making statements of general trends. Sampling error increases when only a single reconstructed sequence is sampled (SD, 1.1 kcal/mol). But even so, a single BI reconstruction results in lower total stability errors than ML and comparable errors to MP, without appreciable systematic bias.

To convert a bias of ΔΔ*G =* −1.5 kcal/mol to a change in folding temperature is difficult, as it depends on the folding entropy and on the heat capacity of the folded and unfolded states. Extensive experiments with point mutations on the thermal stability of bacteriophage T4 lysozyme indicates a change of about 4 °C in the folding temperature for every kcal/mol change in ΔΔ*G* [21]. A bias of −1.5 kcal/mol would then translate to an increase in the folding temperature of approximately 6 °C. While this exact value may be dependent upon the details of the protein and evolutionary models, it is clear that the magnitude of the effect can be significant.

What is the basis behind the tendency of ML and MP to overestimate ancestral-state stability? One clue is in the relatively narrow range of true Δ*G*_Folding_ values as shown in Figure 1. Proteins are generally marginally stable because the pressure to increase stability (minimise Δ*G*_Folding_ and maximise *P*_Target_) is counteracted by the smaller number of highly stable protein sequences versus the large number of less stable sequences [19, 22]. This means that stabilising residues tend to dominate a position over long-term evolution, while the destabilising residues are present but less frequent.

Every location will, given sufficient evolutionary time, be represented by a distribution of amino acids, with the amino acids favourable for important protein properties dominating the distribution. The winner-takes-all nature of the ML and MP approaches will usually assign to the ancestor the most favourable amino acids at each location. Less favourable amino acids that are found in that location with some more limited frequency will be excluded from the reconstructions. As a result, the ancestral proteins will be depleted of these less-favourable residues, with a resulting overestimation of the ancestral protein's properties, in much the same way that majority political parties are overrepresented in democratic assemblies elected by winner-takes-all balloting. These less-favourable residues will be present in the appropriate frequency in BI reconstructions.

Additionally, ML reconstructions will be biased toward the state (here, amino acid) with the highest frequency in the model. For extremely deep nodes in a tree with extremely long branch lengths to the tips, this means that ML would reconstruct a polyalanine ancestor. While no one would believe such a reconstruction, smaller branch lengths would yield more subtle biases. If the most frequent amino acids have a general tendency toward stabilising interactions, the bias in the reconstructions toward these amino acids will provide some bias toward excessive stabilisation. Again, this bias is not present in BI reconstructions.

Note that the bias in ML reconstructions does not depend on the inaccuracy of the substitution model. As is observed, less accurate substitution models do not greatly affect the bias. In fact, substitution models that better represent the different propensities of the various amino acids for different types of locations might have a greater bias toward the most common, that is, most stabilising amino acids at a location. In this case, these more accurate models might result in larger, rather than smaller, biases.

In general, this effect might occur when there are a number of locations in the protein that contribute to a resulting property, and where this property is not optimised by the evolutionary process. In this case, we would expect that some protein locations would have residues that contribute, and some locations would have residues that deduct, from this particular property. And again, when the reconstruction is performed, the less favourable amino acids at each location will be preferentially eliminated, resulting in an overestimation of that property in ancestral sequences. The most obvious example of that type would be some bulk property such as protein stability, as investigated here. But many functional properties might be similarly affected. For instance, binding of a ligand depends upon a number of amino acids at the binding site. The stochastic process of evolution, assuming moderate but not excessive selective pressure, would result in the distribution of functionally favourable and -unfavourable amino acids, with the functionally favourable amino acids excessively dominant in the ancestral reconstructions. As a result, binding affinities (and the potentially related catalytic rates) might be overestimated by ML and MP reconstruction methods.

It is interesting to note that MP seems to have less systematic error in the estimation of ancestral protein stability compared with ML, even though the reconstructions have lower sequence accuracy. Part of the reason might be the tendency of ML to overestimate the number of common amino acids in ancestral proteins. MP might also produce reduced systematic biases as it has a tendency to choose a certain number of less-likely possibilities in different locations. In this way, the increased errors in the MP reconstructions might, to a limited extent, counter the biases produced by its winner-takes-all approach.

We have tried to mimic protein evolution by using evolutionary simulations that contain many of the salient features of real molecular evolution (e.g., population dynamics, interacting locations, nonadditive fitness functions). Nevertheless, they clearly cannot replicate protein thermodynamics or evolutionary dynamics precisely. Although pair-potentials have achieved widespread use and success in the demanding area of protein structure prediction, the stabilities of real biological proteins are only approximated by the pair-potentials used in this study. There may be specific stabilising interactions that are interrupted by reconstruction errors. Changes in the protein backbone are not included in this analysis. Furthermore, we have simulated only one protein structure. A legitimate question is therefore how general the results are expected to be. We expect that the precise details of how functional bias and random error accumulate will depend on the true details of protein energy functions. The proportion of sites that contribute meaningfully to function will vary depending on the details of protein structure and how the protein carries out its functional requirements. Furthermore, epistatic interactions are likely to accumulate differently in different real proteins, and so the time period during which the BI reconstructions are unbiased is also likely to vary.

While the bias present in any specific experimental reconstruction cannot be determined from this simplified analysis, it is clear that there is a strong risk that bias will accumulate for deeper nodes as the uncertainty of reconstruction at each position becomes higher. In many cases, it accumulates quite rapidly for the ML and MP methods, and much more slowly for the BI approach. Although at some divergence levels, the statistical bias toward thermostability in the ML and MP approaches may be overcome by the accumulation of inaccurately reconstructed compensatory pairs, this does not provide a convincing rationale for using these approaches; a stopped clock will be correct twice a day, but is still useless for keeping time.

The fundamental question that needs to be asked in reconstruction experiments is whether the property in question has a tendency to associate with low or high frequency variants at any positions in the protein. It appears to us that this is likely to be the case often enough that BI should always be used rather than ML or MP. For example, ligand binding and catalysis may often depend on key highly stable amino acid interactions and would therefore be better predicted by Bayesian sampling from the posterior. Despite the intuitive appeal of getting a “more correct” sequence with ML, we do not have any clear examples where it is preferable to use ML to reconstruct properties. Concerns over increased variability with BI can be addressed by sequencing three to ten variants, depending on resources.

Even using BI, there may still be sensitivity to the use of incorrect models. The discrepancies between the number of expected and the number of observed errors in the ML and BI approaches indicate that the simple models are doing an imperfect job at representing the complicated reality of our population simulations. We expect that similar discrepancies occur when modelling real protein evolution, and so the accumulation of bias should always be considered as a possible explanation for a given set of results. Since biases affect the ancestral states relative to the extant proteins, they will tend to result in similar parallel trends along different branches of the tree. As a result, signatures of this type of bias include cases where the reconstructed ancestor differs from all or most of its descendants or where parallel trends are observed from ancestors to descendants in many independent branches or taxon groups. While we have not demonstrated that any particular study is incorrect, it is these types of observations that should be carefully confirmed, since they are both extraordinary and mimic reconstruction bias; the simplest (null) explanation is that such cases represent incorrect inference, not true ancestral shifts in function. We expect that the majority of ancestral shifts in function will have occurred once on a single evolutionary lineage, and would therefore be less suspect.

## Materials and Methods

### Protein model.

The goals of these evolutionary simulations require a protein model that is sufficiently accurate to provide interpretable results but sufficiently simple to allow calculations for modestly large population sizes over many generations and many replicates. In previous work, we have performed evolutionary simulations of simplified lattice proteins [23–25]. Here, in order to have a biologically reasonable protein, we chose the native state of a real existent protein, that of the purple acid phosphatase (PDB designation 1QHW [26]), an αβ four-layer sandwich, as it was of reasonable size (300 residues) and compact and contained a variety of secondary structures. The stability of a sequence was measured as the energetic preference for this predesignated native state in thermodynamic competition with an ensemble of misfolded states, while the fitness measured the fraction of time that the protein would be correctly folded.

It is, of course, impossible to completely characterise the entire set of possible unfolded or misfolded structures, so we selected 46 random structures to characterise the distribution of thermodynamic properties of the ensemble of unfolded states. These random structures were chosen from the set of single-strand protein domains of unique topology, between 300 and 399 amino acids in length, based on the CATH database [27]. (The PDB accession codes of the selected structures are listed in Table S2 [28].) For proteins longer than 300 residues, only the first 300 residues were considered. The use of relatively compact decoys to represent alternative conformations of the protein is based on the assumption that, while compact states are not the most numerous alternative states, they are the most likely to be thermodynamically accessible under native-like solvent conditions. Thus, we assumed that it is the competition between folding and misfolding (rather than between folding and unfolding) that is the major selective pressure acting on protein evolution.

A simple contact potential was used to calculate the free energy of the target and 46 random structures [23–25]. The free energy *G(k)* of a sequence {*A*_1_,*A*_2_,…*A*_300_} in structure *k* is given by:


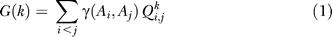


where γ(*A_i_*, *A_j_*) is the contact potential between amino acids *A_i_* and *A_j_* in positions *i* and *j*, and 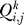 is one if *i* and *j* are in contact in structure *k*, and zero otherwise. Residues were considered to be in contact if their *C*_β_ atoms (*C*_α_ in the case of gly) were within 6 Å of each other. The contact potentials were obtained by Miyazawa and Jernigan, based on a statistical analysis of known protein structures [29]. Due to the nature of their statistical analysis, these potentials represent “potentials of mean force” and implicitly include hydrophobic interactions and other effects of the solvent.

As described above, the energies of the 46 random structures are used to estimate the distribution of energies of a much larger ensemble. The energies of the random structures roughly followed a Gaussian distribution. We modelled the entire set of nonnative structures with a continuous Gaussian energy distribution with mean 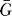 and variance σ^2^, both calculated from the energies of the set of 46 random structures. For protein stability, the quantity that matters is the free energy difference (Δ*G*_Folding_) between the native state and an ensemble of *N* alternative states (modelled with mean 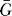 and variance σ^2^), given by


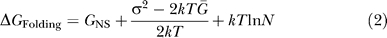


where *kT* is equal to 0.6 kcal/mol. We used a value of 10^160^ for *N*, representing approximately 3.4 conformations available to each amino acid. This value, reasonable in magnitude, was chosen so that an extremely small fraction of random sequences would be stable, yet mutations would be accepted at a reasonable rate during the simulation. *P*_NS_, the probability that the protein is in its native conformation is given by


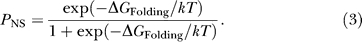


Since protein stability represents *differences* in free energy, it is relatively insensitive to changes in the overall composition of the protein (such as inclusion of more charged groups or hydrophobic residues), which would have a similar impact on all compact conformations.

### Evolution model.

We modelled populations of protein sequences evolving through mutation, selection, replication, and speciation. Mutations occurred at a rate of 0.1 amino acid substitution per sequence per generation, and the relative fitness of each new sequence was computed as the probability of folding into the target state (*P*_NS_, Equation 3). To form the next generation, the frequency of each variant was multiplied by its relative fitness and sampled with replacement. The population size was maintained at 1,000 proteins throughout the simulation.

Prior to beginning each simulation, the population was initiated as 1,000 copies of a single random protein sequence and allowed to reach mutation/selection equilibrium by evolving for 10,000 generations. This allowed the remainder of the simulation to proceed in a nearly neutral manner, with no overall increase in protein stability or overall change in amino acid composition. Speciation events consisted of duplicating the entire population, after which the two population copies mutated and evolved independently from one another. In order to use a biologically reasonable phylogenetic tree, the pattern and timing of speciation events were based on a phylogeny generated from a set of 20 ribonuclease A mammalian gene sequences (listed in Table S3). The tree (shown in Figure S3) was generated using MrBayes [13] and rooted with sequences from chicken, frog, and iguana. Although this tree almost certainly has errors with respect to the true phylogeny and rooting of these species, this is irrelevant to our study since it represents a biologically plausible tree and the true tree for our simulations.

The total length of the tree was 302,864 generations, and 100 replicate evolutionary simulations were performed. After preequilibration, 16 of these simulations were obviously trapped in local maxima with low fitness (*P*_NS_ < 0.9) and were excluded from further simulation and analysis. There were 15 ancestral nodes for each of the 84 remaining simulations, and therefore 1,260 ancestral proteins were simulated and reconstructed. There is some question about what the appropriate “ancestral” sequence actually is. Any of the variants that exist at the moment of speciation might become the apparent ancestor, depending on the stochastic interaction of selection and drift in both populations, although the most frequent variant at that time is most likely to succeed. Since the most frequent variant is also most likely to reflect the properties of future successful substitutions, we chose the most common sequence in the population as the “wild-type.” Subsequent observations of variation in reconstructions indicated that this choice did not have a noticeable impact on the results.

### Phylogenetic analysis.

ML and BI methods of ancestral reconstruction require a model of the substitution process. Standard substitution matrices are based on average processes in biological datasets, and although they are almost certainly suboptimal even for those datasets, we considered it more conservative to use a substitution matrix that is optimised for our sequences to give the ML and BI methods the best chance to work successfully. Using the complete set of sequences obtained in our 84 simulations (representing 84 × 300 = 25,200 sites) and the phylogenetic tree that described their relationship to one another, the optimal (highest log likelihood) reversible substitution matrix with a Γ distribution of rate classes was found. (The software was written taking advantage of the PAL Java library [30].) We thus mimic the situation in real life where the true substitution process is unknown and is instead represented by a computationally convenient Markov chain model that best describes the observed data.

Mesquite [31] and PAML [32] were used to generate reconstructions of all internal nodes for the MP and ML methods, respectively. Locations that were ambiguous in the MP reconstruction were selected at random from the possible options. PAML provides the marginal posterior probability of every amino acid at each location in the ancestral reconstructions, and for the BI “reconstructions” amino acids were selected at random based on these probabilities. For BI, the expected error was estimated using the fact that the probability of correct re-creation of a single location is equal to the sum of the squares of the posterior probabilities.

## Supporting Information

Figure S1 Distribution of Maximum Posterior Probabilities for Reconstructions of the Three Nodes Indicated in Figure S3
For each node, the fraction of all sites that are either reconstructed correctly (solid) or incorrectly (cross-hatched) for a given value of the maximum ML posterior probability for the three different reconstruction methods: ML (red), MP (green), and BI (blue). Note the change in scale in the *y* axis.
(105 KB PDF)

Figure S2 Distribution of Posterior Probabilities for ML Reconstructions of the Three Nodes Indicated in Figure S3
For each node, the fraction of all sites that are either reconstructed correctly (solid) or incorrectly (cross-hatched) for a given value of the posterior probability for four different substitution models as listed in Table S1: optimised (with Γ) (red), WAG + F + Γ [20] (cyan), WAG + Γ (magenta), and WAG (orange). Note the change in scale in the *y* axis.
(117 KB PDF)

Figure S3 The Phylogenetic Tree Used in the Evolution Simulations, Based on a Phylogenetic Reconstruction of 20-y RNase A Gene Sequences The three internal nodes used in Figure 1 are marked.
(88 KB PDF)

Table S1 Average Reconstruction Sequence Accuracy, Average Posterior Probabilities, and Average Thermodynamic Stability Accuracy for ML Reconstructions Produced Using Different Substitution Models “Optimised (with Γ)” refers to the substitution model optimised for the generated sequence data as described in the text. We also used the WAG substitution matrix [20], with default equilibrium frequencies or with equilibrium frequencies estimated based on the simulated data (+F), and without or with gamma-distributed rate variation (+ Γ).
(30 KB DOC)

Table S2 PDB Designation of Structures Used to Form the Random Ensemble of States (84 KB PDF)

Table S3 Ribonuclease Sequences Used to Construct Model Phylogenetic Tree (114 KB PDF)

## Abbreviations

BI: Bayesian inference
ML: maximum likelihood
MP: maximum parsimony
