# Supplementary material for: Assessing the Accuracy of Ancestral Protein Reconstruction Methods
Source: PLoS Comput Biol. 2006 Jun 23;2(6):e69. doi: 10.1371/journal.pcbi.0020069 (PMC1480538; doi:10.1371/journal.pcbi.0020069)

Supplementary Figure 1: Distribution of maximum posterior probabilities for reconstructions of the three nodes indicated in Supplementary Figure S3. For each node, the fraction of all sites that are either reconstructed correctly (solid) or incorrectly (cross-hatched) for a given value of the maximum ML posterior probability for the three different reconstruction methods: ML (red), MP (green), and BI (blue). Note the change in scale in the *y* axis.

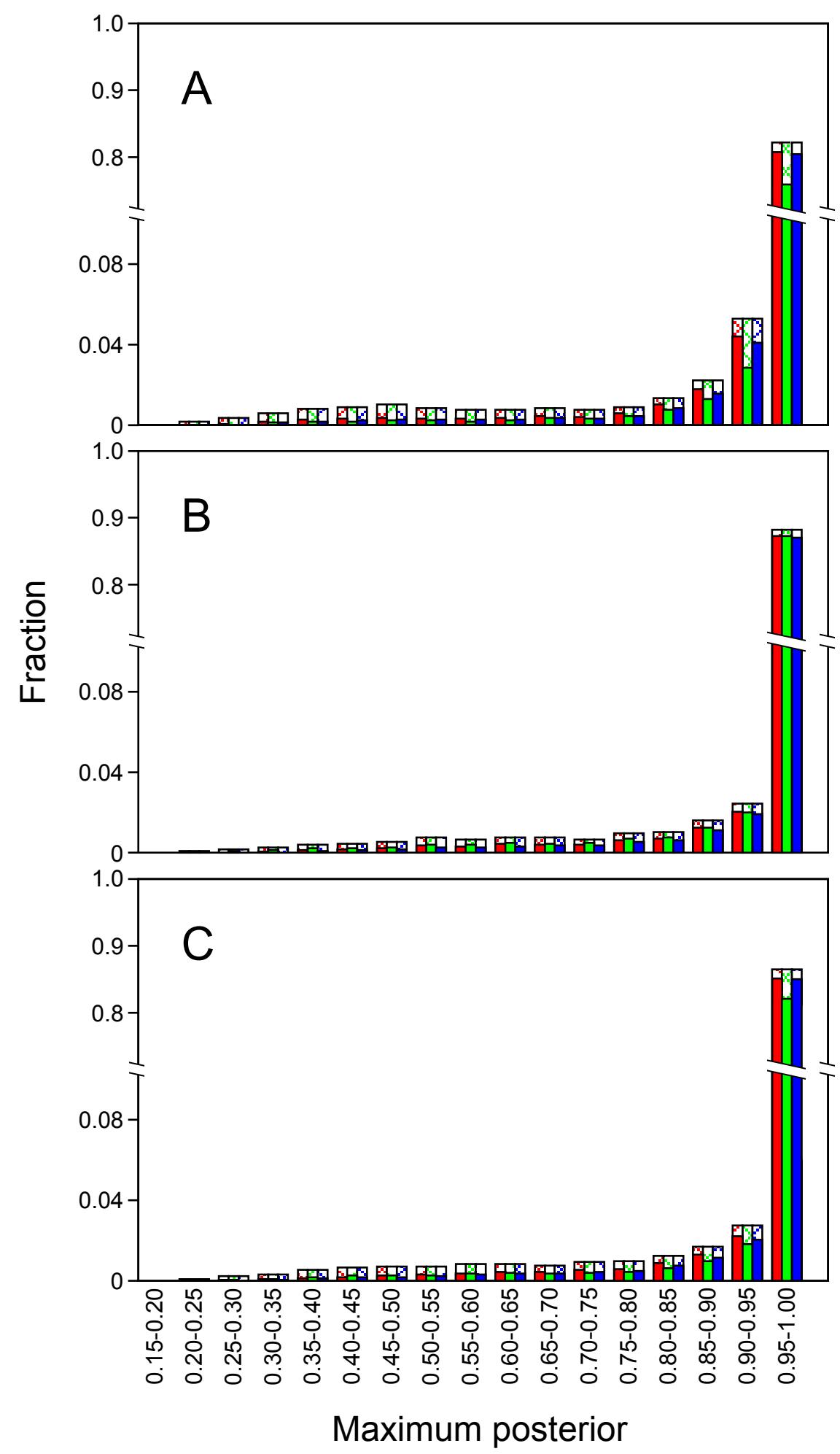

Supplement: Supplementary file 1 [file pcbi.0020069.sg001.pdf]
