# Supplementary material for: Assessing the Accuracy of Ancestral Protein Reconstruction Methods
Source: PLoS Comput Biol. 2006 Jun 23;2(6):e69. doi: 10.1371/journal.pcbi.0020069 (PMC1480538; doi:10.1371/journal.pcbi.0020069)

Supplementary Figure S2: Distribution of posterior probabilities for ML reconstructions of the three nodes indicated in Supplementary Figure S3. For each node, the fraction of all sites that are either reconstructed correctly (solid) or incorrectly (cross-hatched) for a given value of the posterior probability for four different substitution models as listed in Supplementary Table S1: Optimised (with Γ) (red), WAG+F+Γ [20] (cyan), WAG+Γ (magenta), and WAG (orange). Note the change in scale in the *y* axis.

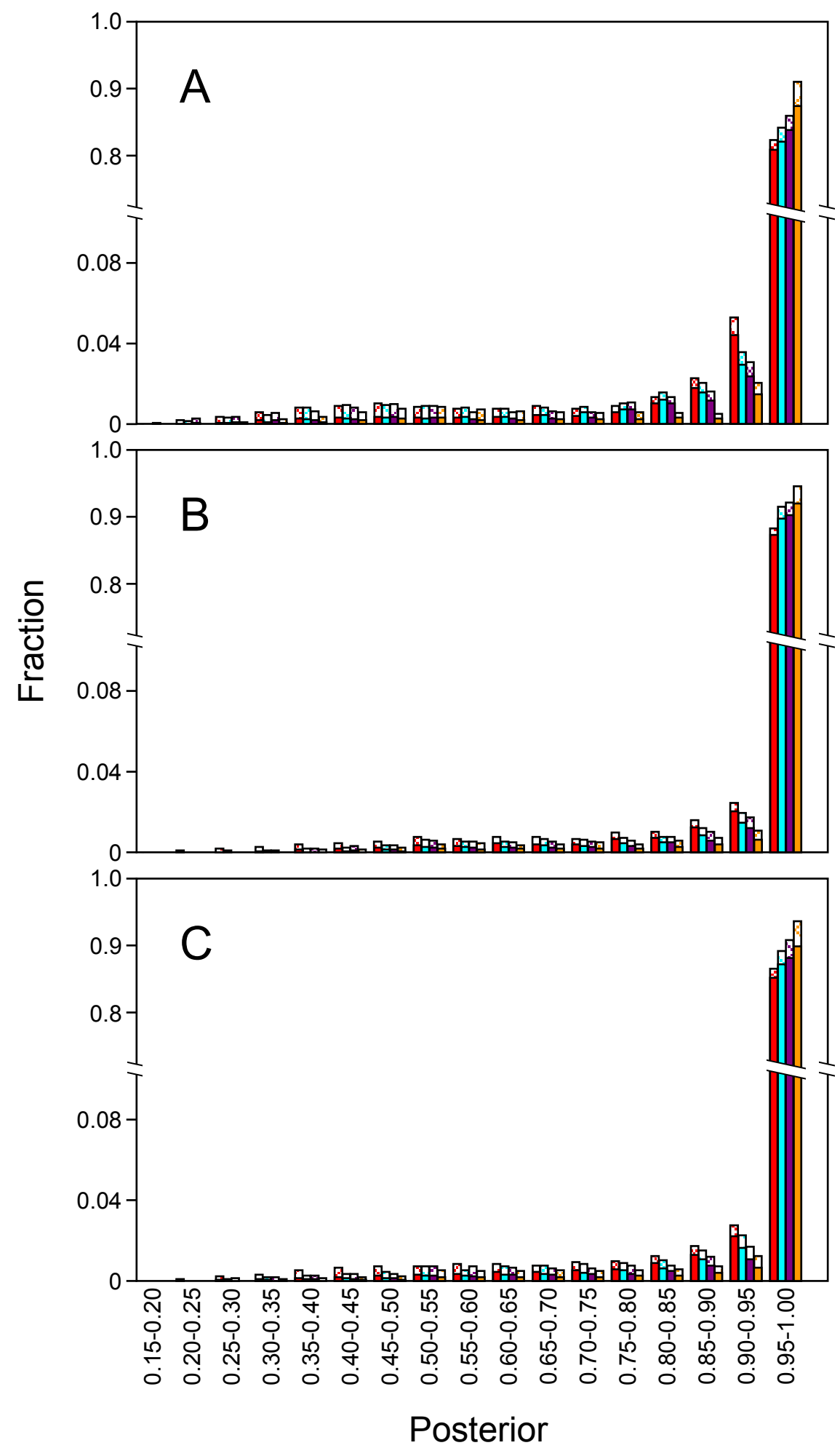

Supplement: Supplementary file 2 [file pcbi.0020069.sg002.pdf]
