# Supplementary material for: Assessing the Accuracy of Ancestral Protein Reconstruction Methods
Source: PLoS Comput Biol. 2006 Jun 23;2(6):e69. doi: 10.1371/journal.pcbi.0020069 (PMC1480538; doi:10.1371/journal.pcbi.0020069)

Supplementary Figure S3: The phylogenetic tree used in the evolution simulations, based on a phylogenetic reconstruction of twenty RNAseA gene sequences. The three internal nodes used in Figure 2 are marked.

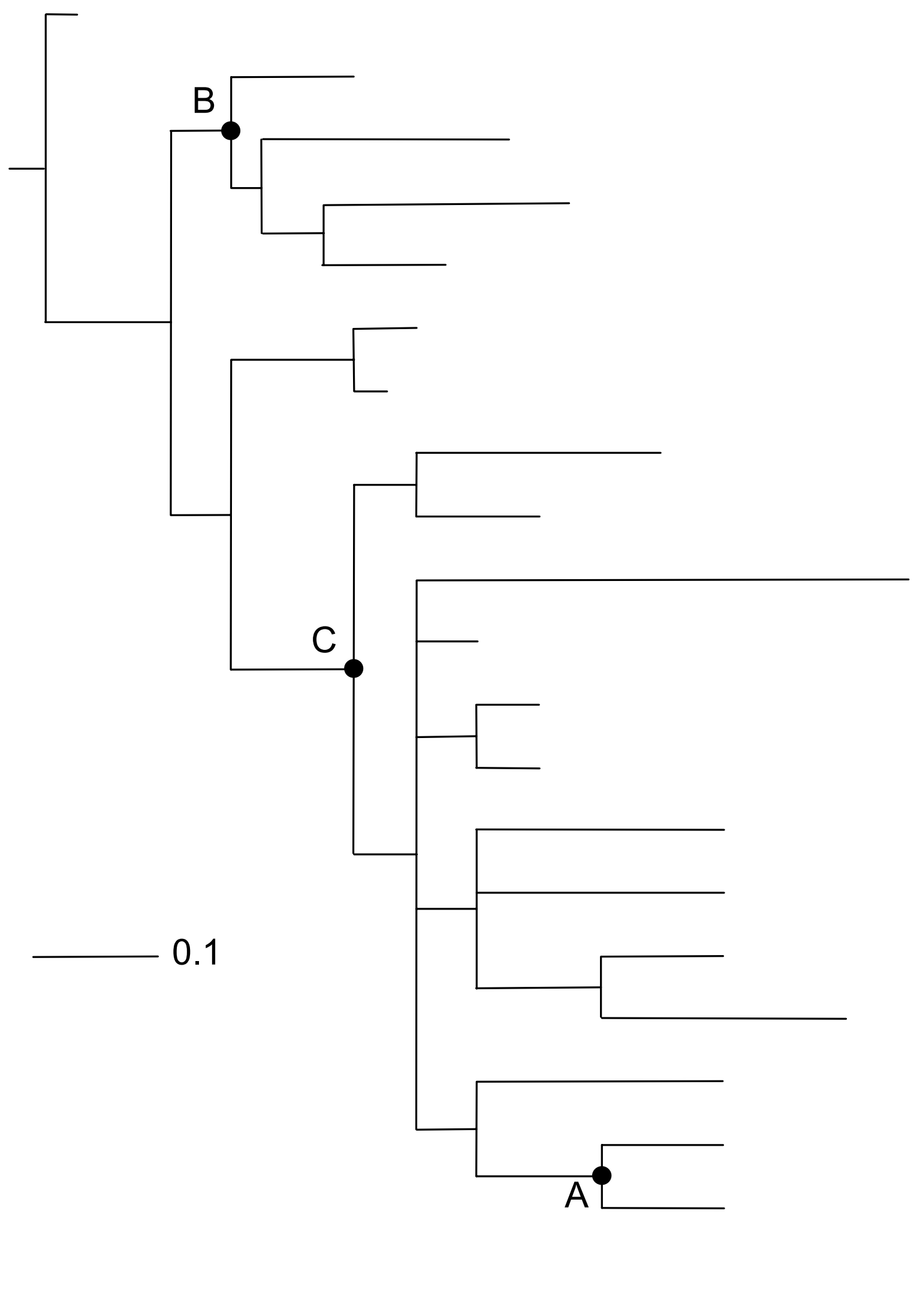

Supplement: Supplementary file 3 [file pcbi.0020069.sg003.pdf]
