# Supplementary material for: Assessing the Accuracy of Ancestral Protein Reconstruction Methods
Source: PLoS Comput Biol. 2006 Jun 23;2(6):e69. doi: 10.1371/journal.pcbi.0020069 (PMC1480538; doi:10.1371/journal.pcbi.0020069)
Supplement: Supplementary file 4 [file pcbi.0020069.st002.pdf]

12as	16vp	1b65	1b7b	1bg2	1bt3	1bwd
1by8	1dmh	1dmw	1dtd	1eg1	1g9n	1ghq
1gq8	1gxr	1hm4	1hyn	1i9z	1idm	1io7
1jfp	1lhp	1m50	1mj5	1mkd	1msv	1nl4
1ofz	1ogq	1oqu	1p8r	1pa1	1pp9	1qoy
1roz	1sde	1sid	1sig	1tdb	1tux	1uby
1uf5	2bbv	3por	3pva			

Supplementary Table S2: PDB designation of structures used to form the random ensemble of states.
