# Supplementary material for: Assessing the Accuracy of Ancestral Protein Reconstruction Methods
Source: PLoS Comput Biol. 2006 Jun 23;2(6):e69. doi: 10.1371/journal.pcbi.0020069 (PMC1480538; doi:10.1371/journal.pcbi.0020069)
Supplement: Supplementary file 5 [file pcbi.0020069.st003.pdf]

Species	Common Name	Genbank designation
Saimiri sciureus	Squirrel monkey	AF449637
Lemur catta	Andlemur	AF449641
Sus scrofa	Pig	AJ005521
Hippopotamus amphibius	Hippopotamus	AJ005522
Phocoena phocoena	Harbour porpoise	AJ005523
Pseudorca crassidens	False killer whale	AJ005524
Glis Glis	Dormouse	AJ005767
Microtus nivalis	Fieldvole	AJ005768
Camelus dromedarius	Camel	AJ006418
Tragulus javanicus	Chevrotain	AJ271297
Atherurus macrourus	Brush-tailed porcupine	AJ535678
Ctenodactylus gundi	Gundi	AJ535679
Sciurus vulgaris	Squirrel	AJ535680
Oryctolagus cuniculus	Rabbit	AJ535681
Artibeus jamaicensis	Fruit bat	AJ535682
Orycteropus afer	Aardvark	AJ535683
Elephas maximus	Elephant	AJ535684
Mus musculus	Mouse	M27814
Homo sapiens	Human	NM_002933
Bos taurus	Ox	X07283

Supplementary Table S3: Ribonuclease sequences used to construct model phylogenetic tree.
