# Supplementary material for: Assessing the Accuracy of Ancestral Protein Reconstruction Methods
Source: PLoS Comput Biol. 2006 Jun 23;2(6):e69. doi: 10.1371/journal.pcbi.0020069 (PMC1480538; doi:10.1371/journal.pcbi.0020069)
Supplement: Supplementary file 6 [file pcbi.0020069.st001.doc]

| **Substitution Model** | **<Sequence Accuracy>** | **<Posterior Probability>** | **<∆∆G>** |
| --- | --- | --- | --- |
| Optimised (with Γ) | 0.937 ± 0.004 | 0.960 | -1.5 ± 0.1 |
| WAG + F + Γ | 0.937 ± 0.004 | 0.969 | -1.3 ± 0.1 |
| WAG + F | 0.937 ± 0.004 | 0.978 | -1.4 ± 0.1 |
| WAG + Γ | 0.931 ± 0.004 | 0.970 | -1.9 ± 0.1 |
| WAG | 0.934 ± 0.004 | 0.980 | -1.7 ± 0.1 |

**Supplementary Table S1**: Average reconstruction sequence accuracy, average posterior probabilities, and average thermodynamic stability accuracy for ML reconstructions produced using different substitution models. ‘Optimised (with Γ)” refers to the substitution model optimised for the generated sequence data as described in the text. We also used the WAG substitution matrix [20], with default equilibrium frequencies or with equilibrium frequencies estimated based on the simulated data (‘+F’), and without or with Gamma-distributed rate variation (‘+ Γ’).
